# Leadership training in emergency medicine: A national survey

**DOI:** 10.1002/aet2.11047

**Published:** 2024-11-21

**Authors:** Shea Palmer, Amanda Rodrigues Amorim Adegboye, Gareth Hooper, Aanika Khan, Caroline Leech, Amanda Moore, Bhupinder Pawar, Ala Szczepura, Chris Turner, Rosie Kneafsey

**Affiliations:** ^1^ School of Healthcare Sciences Cardiff University Cardiff UK; ^2^ Research Centre for Healthcare & Communities Coventry University Coventry UK; ^3^ Royal Borough of Kensington & Chelsea London UK; ^4^ University Hospitals Coventry & Warwickshire NHS Trust Coventry UK; ^5^ UCL Centre for Behaviour Change University College London London UK; ^6^ School of Health Coventry University Coventry UK; ^7^ Centre for Care Excellence Coventry University and University Hospitals Coventry & Warwickshire NHS Trust Coventry UK

**Keywords:** education, emergency medicine, innovation, leadership, learning, specialty training, surveys and questionnaires

## Abstract

**Background:**

Emergency medicine (EM) is a uniquely stressful environment in which leadership training could improve individual and team performance, patient outcomes, well‐being, and EM career intentions. The primary aim was to evaluate EM‐specific leadership training (EMLeaders) compared to no leadership training. A secondary comparison was with other forms of leadership training.

**Methods:**

An online survey was distributed to Royal College of Emergency Medicine (RCEM) members in England. Three groups were recruited: those who reported receiving EMLeaders training, no training, and other training. Information was collected on group demographics, job roles, responses to 14 leadership knowledge and skills items, well‐being at work, and EM career intentions.

**Results:**

A total of 417 responders (177 EMLeaders, 148 no training, 92 other training) were largely representative of RCEM members, although the EMLeaders group were at less senior career grades. Although all groups provided generally positive responses, EMLeaders demonstrated more positive ratings for seven of 14 leadership items relative to no training (all *p* < 0.05): knowledge about clinical leadership, application of clinical leadership, empowerment to make decisions, managing the emergency department environment, ability to influence the EM environment, confidence in leadership, and confidence in facilitating teams. The other training group demonstrated superior ratings for five of seven of the same items, except empowerment to make decisions and ability to influence the EM environment. Direct comparison of EMLeaders with other training identified ability to influence the EM environment as a unique benefit of EMLeaders (*p* < 0.05), while knowledge about clinical leadership favored other training (*p* < 0.05).

**Conclusions:**

EMLeaders improved many aspects of perceived leadership knowledge and skills, but there was little evidence of impact on well‐being or EM career intentions. EMLeaders particularly appears to enhance perceived ability to influence the EM environment. Considering that the EMLeaders group were generally earlier in their career, the findings are promising and can inform the refinement of future EM‐specific training.

## INTRODUCTION

Effective leadership skills within the emergency department (ED) can improve patient‐related outcomes, including care quality, safety, and mortality, as summarized by Larsen and colleagues,^1^ and also health professionals’ well‐being. Indeed, during the COVID‐19 pandemic, a range of teamwork and leadership attributes were associated with lower clinician burnout.^2^ However, many health care professionals feel inadequately prepared for leadership roles.^1^ Given the complexity of leadership in emergency medicine (EM), the importance of explicitly developing leadership skills has been identified.^3^ However, the most effective form of leadership training for EM professionals remains undetermined. A systematic review of leadership development in medicine (not specific to EM) found that active learning (undertaking projects, mentorship, and individualized goals) was more important than specific curriculum content.^4^ Hansen and colleagues^5^ found that two forms of brief (60 min) online leadership training were equally effective for EM and obstetrics/gynecology residents. There is clearly a lack of evidence in this area.

EM professionals face particular challenges due to the intense and often chaotic working environment and unpredictable caseload. 34% of EM trainees in the United Kingdom are at high risk of burnout, with 73% rating their workload as heavy/very heavy, both figures being the highest of all specialties.^6^ A burnout rate of 76.1% of EM residents was identified in the United States and was linked with reduced patient care quality, reduced empathy, and more clinical errors.^7^ Burnout is also associated with reduced professionalism and job satisfaction and increased intentions to leave the profession.^7^ However, it is clear that there are multiple factors associated with EM staff retention.^8^ A recent qualitative study found effective leadership to be closely associated with EM staff well‐being and retention,^9^ with development and refinement of leadership skills identified as a potentially important driver of positive change in this area.

An EM‐specific leadership program (termed EMLeaders)^10^ has been developed to improve personal and team leadership skills. It was jointly created by the Royal College of Emergency Medicine (RCEM), Health Education England (HEE), and National Health Service (NHS) Improvement (now incorporated into NHS England), and launched in April 2018. The program focuses on knowledge and application of leadership theory, managing difficult decisions, handling challenging and conflict situations, and creating an EM learning culture. It is offered to medical and nonmedical professionals working in EM, regardless of seniority. Originally designed for face‐to‐face delivery, the program has evolved, moving to online delivery during the COVID‐19 pandemic and adding new e‐learning modules and communities of practice. At the time of this research the program included on‐the‐job “shop‐floor” training events (70% of the program), self‐directed learning (20%), and formal learning (10%). We have recently published a qualitative evaluation of the EMLeaders program,^11^ which provided rich evidence of positive effects on leadership knowledge and skills. The EM‐specific nature of the training was particularly valued, although measures to more effectively embed the program within practice were highlighted.^11^ The current paper presents quantitative data to complement the published qualitative findings, broadening the commissioned evaluation by contextualizing the reported benefits of EM‐specific training through comparison with other groups.

This study had a primary aim of determining the relative effectiveness of EM‐specific leadership training (EMLeaders training) compared to no training on self‐reported leadership knowledge and skills, well‐being at work, and future EM career intentions. A secondary aim was to compare EMLeaders and no training with other forms of leadership training (other training). It is the first study to quantitatively evaluate specialty‐specific leadership training and aims to inform future training design and delivery.

## METHODS

Ethical approval was received from Coventry University Ethics Service (Reference P124919) and the research is reported in accordance with the Checklist for Reporting of Survey Studies (CROSS).^12^ A bespoke cross‐sectional online survey explored the impacts of leadership training on key aspects of EM working. Three participant groups were recruited: those who self‐identified as having received EMLeaders training, no training, and other training in leadership.

A copy of the final survey is provided in Data S1. Survey items and response options were developed and refined in an iterative process with relevant experts: research team members (including two EM consultants—CT and CL) and RCEM and HEE staff. Rather than having a specific theoretical basis, survey items addressed the stated aims of the EMLeaders program (available at https://rcem.ac.uk/em‐leaders‐programme/, accessed July 12, 2023), which were: “to ensure that those within the ED are: more knowledgeable about clinical leadership and how to apply it on the shop floor; empowered to make decisions in the workplace and manage the challenging environment of the emergency department; supported by the School leadership faculty with their learning and are enabled to feedback personal experiences or concerns.” Additional items were suggested by experts based on their experience of leadership training and operational leadership in EM, leading to 14 primary survey items. These items were considered generic and covered perceived knowledge about clinical leadership, application of clinical leadership, empowerment to make decisions, managing the ED environment, being enabled to feedback personal experiences or concerns, ability to influence the EM environment, confidence in decision making, confidence in leadership, confidence in facilitating teams, positive well‐being, enthusiasm about pursuing a career in EM, listening effectively, recognizing demands, and adapting to demands. The questions on well‐being and EM career intentions were included to inform discussion about potential longer term effects of leadership training. Responders who had received leadership training (EMLeaders or other training) completed two additional items, addressing how supported they felt with their learning and development, and how likely they were to recommend their training to their colleagues. Items were presented as positively worded statements (e.g., “I am knowledgeable about clinical leadership”) and responders were asked to rate their agreement using a 6‐item Likert scale (from 1 = strongly agree to 6 = strongly disagree). Demographic (seven questions) and professional background (five questions) items mirrored previous RCEM membership surveys, allowing direct comparison.

As the primary focus was on evaluating EMLeaders, five additional questions specific to that program were added, including the region of England, year started, type of training, modules completed, and whether they had decided to disengage from further EMLeaders training. Four open‐ended questions were also included and those qualitative findings have been reported separately.^11^

The survey was hosted on Joint Information Systems Committee (JISC) online surveys and was thoroughly piloted by the same group of experts (research team and RCEM and HEE staff) before being advertised to potential participants. Piloting focused on technical progression through the online survey and therefore using the same group of experts was considered acceptable. Item logic ensured relevance and all items were compulsory to ensure data completeness. The survey incorporated study information, a privacy statement, and explicit informed consent. Participants could withdraw consent simply by closing their internet browser, and data completed to that point were deleted. All surveys were completed anonymously. Study information, a request to participate, and link to the online survey were distributed to a convenience sample, comprising all RCEM members, including doctors, physician associates, advanced care practitioners, and other health professionals (*n* = 9212). There were no specific exclusion criteria. All RCEM members were approached, not just trainees, so that data could be generated about all forms of leadership training, including that undertaken historically by more experienced professionals. An initial email invitation was sent the week beginning December 20, 2021, and a reminder was sent the week beginning January 10, 2022. The survey closed on January 31, 2022. A formal sample size calculation was not conducted as the likely variability in response data was unknown. However, projected response rates of 5% (*n* = 461) and 10% (*n* = 921) were estimated (with 95% confidence) to provide margins of error of 4.45% and 3.06%, respectively, either of which was considered acceptable.

### Data analysis

Potential multiple participation by the same individuals was checked using demographic data and occupational details, with no instances identified. Demographics and professional background items were analyzed using descriptive statistics (numbers and proportions) by group (EMLeaders training, no training, and other training) and as a total cohort. This was compared against RCEM membership survey demographic data (received May 10, 2022). Data for the main survey items were presented using medians (and interquartile range [IQR]). Mean values have also been reported to aid with interpretation. The statistical significance of differences between groups was explored using nonparametric Pearson chi‐square, Kruskal–Wallis, and post hoc Mann–Whitney tests as data was nominal/ordinal and nonnormally distributed (Kolomogrov–Smirnov test *p* > 0.05). Data specific to EMLeaders were presented descriptively using numbers and proportions. Analyses were conducted using IBM SPSS Statistics 27. There were no missing data as all data fields were compulsory.

## RESULTS

A total of 447 responders accessed the survey, with 417 complete responses (93.3% completion rate). This represents 4.5% (417/9212) of RCEM members and provided an acceptable estimated margin of error (with 95% confidence) of 4.69%. A total of 177 responders received EMLeaders training, 148 no training, and 92 other training. Table 1 presents the demographic data. Responders were broadly representative of RCEM members in terms of career grade, ethnicity, sex, and disability, with some minor deviations (e.g., a larger proportion of specialty and associate specialist [SAS] doctors and a lower proportion of advanced care practitioners responded to our survey and a higher proportion reported a disability).

**TABLE 1:** Survey participant demographics.

Question/response	RCEM membership (%)	EMLeaders training (*n* = 177)	No training (*n* = 148)	Other training (*n* = 92)	Total (*n* = 417)
“Are you currently working in emergency medicine (EM)?” *p* = 0.320					
Yes	N/A	163 (92.1)	137 (92.6)	89 (96.7)	389 (93.3)
No	N/A	14 (7.9)	11 (7.4)	3 (3.3)	28 (6.7)
“Please select which career grade applies to you” *p* < 0.001*					
Consultant	32.4	58 (32.8)	40 (27)	46 (50)	144 (34.5)
Locum consultant	2.6	4 (2.3)	5 (3.4)	5 (5.4)	14 (3.4)
SAS doctor (staff grade, associate specialist, and specialty doctors)	12.6	10 (5.6)	50 (33.8)	18 (19.6)	78 (18.7)
Trainee ST1	6.8	7 (4)	10 (6.8)	0	17 (4.1)
Trainee ST2	6.8	18 (10.2)	4 (2.7)	3 (3.3)	25 (6)
Trainee ST3	7.5	13 (7.3)	6 (4.1)	2 (2.2)	21 (5)
Trainee ST4	3.4	17 (9.6)	3 (2)	0	20 (4.8)
Trainee ST5	5.1	19 (10.7)	3 (2)	1 (1.1)	23 (5.5)
Trainee ST6	6.2	20 (11.3)	4 (2.7)	1 (1.1)	25 (6)
Physician associate	1.2	0	1 (0.7)	0	1 (0.2)
Advanced care practitioner	15.4	8 (4.5)	15 (10.1)	13 (14.1)	36 (8.6)
Other	N/A	3 (1.7)	7 (4.7)	3 (3.3)	13 (3.1)
“Have you been involved with supporting participants on EMLeaders training events?” *p* < 0.001*					
Yes	N/A	30 (16.9)	3 (2)	3 (3.3)	36 (8.6)
No	N/A	147 (83.1)	145 (98)	89 (96.7)	381 (91.4)
“Have you undertaken EMLeaders training events?” *p* = N/A					
Yes	N/A	177 (100)	0	0	177 (42.4)
No	N/A	0	148 (100)	92 (100)	240 (57.6)
“Have you undertaken other external leadership training?” *p* = N/A					
Yes	N/A	N/A	0	92 (100)	92/240 (38.3)
No	N/A	N/A	148 (100)	0	148/240 (61.7)
“What ethnic group do you identify as?” *p* = 0.300					
Asian/Asian British	27.8	39 (22.2)	46 (31.7)	22 (23.9)	107 (25.9)
Black/African/Caribbean/Black British	6.6	6 (3.4)	6 (4.1)	7 (7.6)	19 (4.6)
Mixed/multiple ethnic groups	3.0	4 (2.3)	2 (1.4)	2 (2.2)	8 (1.9)
Other ethnic group	5.5	12 (6.8)	12 (8.3)	3 (3.3)	27 (6.5)
Prefer not to say	5.2	6 (3.4)	8 (5.5)	6 (6.5)	20 (4.8)
White	51.9	109 (61.9)	71 (49)	52 (56.5)	232 (56.2)
“What is your sex?” *p* = 0.275					
Male	61.2	91 (51.7)	89 (60.5)	57 (64)	237 (57.5)
Female	38.3	78 (44.3)	51 (34.7)	29 (32.6)	158 (38.3)
Prefer not to say	0.5	7 (4)	7 (4.8)	3 (3.4)	17 (4.1)
“Do you consider yourself to have a seen or unseen disability?” *p* = 0.111					
Yes	4.9	15 (8.6)	9 (6.1)	15 (16.5)	39 (9.5)
No	94.9	153 (87.9)	134 (91.2)	73 (80.2)	360 (87.4)
Prefer not to say	0.2	6 (3.4)	4 (2.7)	3 (3.3)	13 (3.2)

*Note*: Data are reported as *n* (%). RCEM membership data has been reported to assist with judgments about representativeness of survey responders. [Please note that responses to the RCEM membership survey were optional and the number of responses therefore varied from a maximum of 7291 for “Please select which career grade applies to you” to a minimum of 5588 for “Do you consider yourself to have a seen or unseen disability?” A pragmatic decision was therefore made to only present the % figures for the responses to each question.] Please note that some follow‐up questions relating to ethnicity, gender, and disability have not been reported here in the interests of being succinct, but these data are available in Data S2. SAS doctors are senior doctors in permanent posts with at least 4 years of postgraduate training, two of which have been in EM; STs are resident doctors undergoing specialty training in EM; physician associates are non–medical health care professionals who support doctors in patient assessment and management; advanced care practitioners are normally nurses or allied health professionals with advanced training and skills in EM.

Abbreviations: EMLeaders, EM‐specific leadership training; N/A, not applicable; RCEM, Royal College of Emergency Medicine; SAS, specialty and associate specialist; ST, specialty trainee.

* Statistically significant difference between groups (Pearson chi‐square, *p* < 0.05).

There were statistically significant differences between groups in the proportion of responders in different career grades (*p* < 0.05). A slightly higher proportion of those who received other training were working at consultant level. Those who received EMLeaders training were more likely to be at specialty trainee (ST; ST2–ST6) level. Unsurprisingly, those who had received EMLeaders training were more likely to be supporting other participants on EMLeaders training events (*p* < 0.05).

Table 1 suggests that there were no statistically significant differences between groups on the basis of ethnic group, sex, or disability (all *p* > 0.05). However, there were statistically significant differences at a more granular level for ethnicity and disability categories (both *p* < 0.05; Data S2). For example, those identifying as White English/Welsh/Scottish/Northern Irish/British were more likely to have received EMLeaders training. And higher proportions of people in the other training group described their disability or impairment as “mental health,” “physical,” “prefer not to say,” or “other.”

A list of the leadership courses identified by other training survey responders is available in Data S3. It is clear that the nature and scope of these courses was extremely variable. One participant (number 32) identified “EM leadership modules (RCEM)—HEE online modules” despite classifying themselves as belonging to the other training group.

Table 2 presents the median (IQR) ratings (1 = strongly agree to 6 = strongly disagree) for each of the 14 main leadership items. The mean ratings are included to assist with interpretation. Participants in all groups were generally positively disposed to the statements, with median ratings of 2 (moderately agree) in almost all cases. The only exceptions were in the no training group for “I am knowledgeable about clinical leadership” and “I am positive about my ability to influence the EM work environment,” with median ratings of 3 (“slightly agree”). There were some differences in IQR values.

**TABLE 2:** Median (IQR) ratings for each leadership knowledge and skills statement answered by all three groups.

Survey statement	EMLeaders training (*n* = 177)	No training (*n* = 148)	Other training (*n* = 92)	*p*‐value for between group differences
I am knowledgeable about clinical leadership	2 (2, 3) $\bar{x}$ 2.25	3 (2, 3) $\bar{x}$ 2.80	2 (1, 2) $\bar{x}$ 2.08	<0.001* ^,^ ** ^,^ *** ^,^ ^****^
I know how to apply clinical leadership on the shop floor	2 (2, 3) $\bar{x}$ 2.15	2 (2, 3) $\bar{x}$ 2.69	2 (1, 2) $\bar{x}$ 2.02	<0.001* ^,^ ** ^,^ ***
I am empowered to make decisions in the workplace	2 (1, 2) $\bar{x}$ 2.04	2 (2, 3) $\bar{x}$ 2.57	2 (1, 3) $\bar{x}$ 2.32	0.002* ^,^ **
I can manage the challenging environment of the ED	2 (1, 2) $\bar{x}$ 1.98	2 (1.75, 3) $\bar{x}$ 2.33	2 (1, 2) $\bar{x}$ 2.07	0.019* ^,^ ** ^,^ ***
I am enabled to feedback personal experiences or concerns	2 (2, 3) $\bar{x}$ 2.34	2 (2, 3) $\bar{x}$ 2.54	2 (1, 3) $\bar{x}$ 2.27	0.244
I am positive about my ability to influence the EM work environment	2 (2, 3) $\bar{x}$ 2.36	3 (2, 3) $\bar{x}$ 2.77	2 (2, 3) $\bar{x}$ 2.76	0.034* ^,^ ** ^,^ ^****^
I am confident in my decision making	2 (2, 2) $\bar{x}$ 2.01	2 (1.75, 3) $\bar{x}$ 2.15	2 (1, 2) $\bar{x}$ 1.89	0.087
I am confident in my leadership	2 (2, 3) $\bar{x}$ 2.13	2 (2, 3) $\bar{x}$ 2.47	2 (1, 2) $\bar{x}$ 1.99	<0.001* ^,^ ** ^,^ ***
I am confident in facilitating teams	2 (2, 3) $\bar{x}$ 2.08	2 (2, 3) $\bar{x}$ 2.36	2 (1, 2) $\bar{x}$ 1.95	0.002* ^,^ ** ^,^ ***
I have positive well‐being at work	2 (2, 3) $\bar{x}$ 2.36	2 (2, 4) $\bar{x}$ 2.80	2 (2, 3) $\bar{x}$ 2.71	0.059
I am enthusiastic about pursuing a career in EM	2 (1, 3) $\bar{x}$ 2.08	2 (1, 3) $\bar{x}$ 2.21	2 (1, 3) $\bar{x}$ 2.43	0.119
I listen effectively to other people within the ED	2 (1, 2) $\bar{x}$ 1.77	2 (1, 2) $\bar{x}$ 1.78	2 (1, 2) $\bar{x}$ 1.79	0.984
I can recognize the differing demands within the ED	2 (1, 2) $\bar{x}$ 1.79	2 (1, 2) $\bar{x}$ 1.84	2 (1, 2) $\bar{x}$ 1.67	0.143
I can adapt to the differing demands within the ED	2 (1, 2) $\bar{x}$ 1.98	2 (1, 2) $\bar{x}$ 1.99	2 (1, 2) $\bar{x}$ 1.84	0.206

*Note*: The mean values $\bar{x}$ have also been reported to aid interpretation of the direction of any differences between groups. Response categories were: 1 = strongly agree, 2 = moderately agree, 3 = slightly agree, 4 = slightly disagree, 5 = moderately disagree, 6 = strongly disagree.

Abbreviation: EMLeaders, EM‐specific leadership training.

* Statistically significant difference between groups (Kruskal–Wallis test, *p* < 0.05). All other *p*‐values relate to statistical comparison between all three groups (Kruskal–Wallis test).

** Statistically significant difference between EMLeaders and no training (Mann–Whitney test, *p* < 0.05).

*** Statistically significant difference between other training and no training (Mann–Whitney test, *p* < 0.05).

^****^ Statistically significant difference between EMLeaders and other training (Mann–Whitney test, *p* < 0.05).

Statistically significant differences were evident between groups for seven of the 14 leadership statements (Table 2, all *p* < 0.05). For all seven of these statements, those who received EMLeaders responded more positively than those who had received no training (all *p* < 0.05). This was also true for five of the same seven statements for those receiving other training (*p* < 0.05). In only two cases were there statistically significant differences between EMLeaders training and other training (both *p* < 0.05). In the first (“I am knowledgeable about clinical leadership”), mean ratings favored other training but in the second (“I am positive about my ability to influence the EM work environment”) mean ratings favored EMLeaders. There were no statistically significant differences between groups for the other seven statements in Table 2, suggesting that leadership training (of any kind) did not influence perceptions of those aspects.

Two statements were rated only by those who undertook either EMLeaders training or other training. There were no differences between groups, with median ratings of 2 (“moderately agree”) for both questions, as follows. “I am supported by [the HEE EM school faculty/colleagues] with my learning and development as a leader,” EMLeaders 2 (2, 3), $\bar{x}$ 2.47, other 2 (1.75, 3), $\bar{x}$ 2.41; *p* = 0.725; “I would recommend the [EMLeaders/external leadership] training that I undertook to my peers,” EMLeaders 2 (1, 3), $\bar{x}$ 2.11, other = 2 (1, 3), $\bar{x}$ 2.26; *p* = 0.317.

Items specific to EMLeaders are summarized in Data S4. Responses were received from all regions, with a dip in engagement during 2020, coinciding with the COVID‐19 pandemic. Engagement with the program was variable. For example, participants undertook a mean of 3.9 modules (696 modules/177 participants) but a sizeable proportion (12.4%) indicated that they had not undertaken any modules. These participants instead engaged with EMLeaders via the alternative learning opportunities. The main mode of participation was via the e‐learning modules (72.9% of responders), followed by face‐to‐face study days (63.3%). Only 13% had participated in the communities of practice. Engagement with the three “core” modules was higher (76.3% for “leading self” and “leading teams” and 58.8% for “leading systems”) than for optional modules. “Leading strategy” (previously called “leading evaluation”) was least frequently undertaken (16.4% of responders). A total of 11.3% had decided not to engage in further EMLeaders training, although reasons for this were not explored.

## DISCUSSION

All three groups were generally positive about their self‐perceived leadership knowledge and skills, well‐being at work, and EM career intentions. Relative to no training, those who received EMLeaders training had more positive scores on seven of 14 items. Other training had more positive scores on five of 14 items. However, the magnitude of differences was very small. Direct head‐to‐head comparison of EMLeaders versus other training found that EMLeaders was superior in one item (“I am positive about my ability to influence the EM work environment”), this being a key program aim. Other training was superior to EMLeaders in one other item (“I am knowledgeable about clinical leadership”). Leadership training (of any kind) therefore seems to be effective, with slight differences depending on the type of training received. Those who received both forms of leadership training were equally positive about the support received and recommending training to their peers. However, there are potential confounders to consider.

Firstly, the relative clinical and leadership experience of responders in each group should be considered. For example, the proportion of EM (ST1–ST6) was much higher for EMLeaders (53.1%) compared to other training (7.6%) and no training (20.3%). ST doctors are on a formal training pathway to consultant; therefore, EMLeaders was specifically championed with this group. At the other end of the career grade continuum, 55.4% of the other training group were at consultant or locum consultant grade, compared to 35.1% and 30.4% of the EMLeaders and no training groups, respectively. As EMLeaders is relatively new, many consultants are likely to have received other forms of leadership training historically and to have had more opportunities to apply their learning in a leadership position than EMLeaders trainees. The proportion of SAS doctors was much smaller in the EMLeaders (5.6%) group compared to other training (19.6%) and no training (33.8%). SAS doctors are usually experienced doctors in specialist EM posts not on a formal training pathway to consultant. The combined impact of these observations suggests that those who had received EMLeaders may have been less experienced in EM than the other groups. Given this, the positivity of responses to survey items related to perceived leadership knowledge and skills in the EMLeaders group is likely to indicate positive outcomes in favor of EM‐specific leadership training. However, this interpretation cannot be definitive in the absence of appropriately controlled trial evidence.

Further potential confounders relate to the content, duration, mode of delivery, and recency of training. For example, those receiving leadership training earlier in their career are more likely to have received such training face‐to‐face, with less focus on EM and integration into practice. The evidence suggests that Other training was highly variable in nature and scope (Data S3), although it should be noted that engagement with EMLeaders was also variable. For example, many EMLeaders responders reported that they had only engaged with e‐module content, predominantly the three core modules. Those who received leadership training more recently, such as those receiving EMLeaders, may have been more able (or more likely) to attribute their leadership knowledge and skills to the training received. Others may have developed leadership knowledge and skills through informal and experiential learning or via leadership training that may have been less explicitly embedded within other formal learning scenarios. These issues complicate the observed relationships between training and the rating of survey items.

The survey findings suggested that leadership training (of any kind) seemed to have little impact on well‐being or EM career intentions. This is contrary to evidence that leadership attributes were associated with lower burnout^2^ and improved well‐being and retention of EM staff.^9^ Our qualitative evaluation of the EMLeaders program suggested improved role satisfaction and that trainees felt valued and connected, with the potential for this to enhance retention.^11^ Given this alternative evidence, it should be acknowledged that the observed lack of effect on perceived well‐being and EM career intentions in the current survey is based on just two unvalidated questions (“I have positive well‐being at work” and “I am enthusiastic about pursuing a career in EM”), and a more nuanced evaluation of these concepts may have been required. Both items were actually rated positively by all groups (median “moderately agree”), so there may have been little scope for further improvement. The EMLeaders program was also relatively new, and recipients may not yet have had an opportunity to adequately apply their learning within practice. An alternative explanation is that the results simply reflect the challenges of the EM environment, including staffing and resource issues and patient acuity. Indeed, Darbyshire and colleagues^8^ have demonstrated that retention of doctors in EM is influenced by complex interactions between many different interlinked factors.

It should be recognized that most of the EMLeaders training evaluated in this study was delivered during the COVID‐19 pandemic when the EM working environment was particularly challenging and face‐to‐face learning was limited, potentially hampering learner experience. Indeed, EMLeaders was originally designed for face‐to‐face delivery and was adapted for online delivery because of the pandemic. The positive results observed under such adverse conditions may indicate potential for even greater effectiveness under more optimal learning conditions. An example of a key leadership skill that might be more difficult to develop online is communication. Chalupnik and Atkins^13^ evaluated the language used by ST doctors in simulated EM scenarios and how this related to leadership performance and the success of teams. High performers used more indirect requests and supportive language than less successful trainees. Communication is therefore an important part of leadership and is more likely to lend itself to face‐to‐face or simulation‐based development. The active learning strategies identified as most effective for medical leadership development^4^ are also likely to require face‐to‐face delivery and time to embed into practice. It would therefore be useful to conduct a further future evaluation of EMLeaders in the absence of restrictions to face‐to‐face learning.

Our study suggested slight differences in the type of training received (or indeed the likelihood of no training), depending on a range of protected characteristics, including disability and ethnicity. People reporting specific types of disability or impairment, such as “mental health” or “physical” were slightly more likely to have received other training. The relevance of this is difficult to interpret but it could indicate appropriate adjustments and personalization of training. There is a lack of UK data, but in the United States, women and racial/ethnic groups underrepresented in medicine were less likely to hold leadership positions and had lower academic ranks in academic EDs.^14^ Those in positions to influence access to leadership training should be vigilant in addressing inequalities.

There are many opportunities for future research in this area, including evaluation of leadership knowledge and skills from other perspectives (such as patients and colleagues). It would also be useful to evaluate whether leadership training is more likely to benefit junior or more senior colleagues, given that those completing EMLeaders in the present study tended to be less experienced. Finally, there was no evidence that aspects related to communication (feeding back and listening), confidence in decision making, and adaptive leadership (recognizing and adapting to demands) were improved following leadership training, yet such skills are important in the ED.^15^ Coaching has demonstrated potential to enhance a wide range of outcomes, including listening skills, well‐being, job satisfaction, and resilience.^16^, ^17^, ^18^, ^19^ Future research could further evaluate the effectiveness of integrating coaching into EM leadership training.

## LIMITATIONS

Key strengths of the current research include that it is the first evaluation of different types of leadership training in EM, the relatively large sample (*n* = 447), and its representativeness of the RCEM membership. Although representative, the survey was distributed across a holiday period, and this may have negatively impacted the response rate. For example, Roberts and colleagues^20^ achieved a much higher response of 1686 EM doctors in a three‐part longitudinal survey of psychological distress during the COVID‐19 pandemic, although recruitment was across the United Kingdom and Ireland and not just England. By their nature, cross‐sectional surveys have limitations, including self‐report, inability to determine cause–effect relationships, responder bias, recall bias (particularly for the other training group), responses being affected by social desirability or acquiescence, and confounders. Importantly, for the current investigation, this includes variability in the nature and scope of other training and variability in the level of engagement with EMLeaders training. This prevents meaningful comparisons between different forms of leadership training, although the comparisons with no training probably remain largely valid. Another important limitation is the lack of specific theoretical basis for the survey. An assessment framework such as that proposed by Rosenman and colleagues^21^ for assessing team leadership in EM may have been appropriate. Equally, well‐being and EM career intentions warrant more nuanced evaluation in future research.

## CONCLUSIONS

In conclusion, leadership training (of any kind) improved many aspects of perceived leadership knowledge and skills, but there was little evidence of effects on self‐reported well‐being or emergency medicine career intentions. Emergency medicine–specific leadership training may enhance perceived ability to influence the emergency medicine environment.

## AUTHOR CONTRIBUTIONS

Shea Palmer, Amanda Rodrigues Amorim Adegboye, Caroline Leech, Ala Szczepura, Chris Turner, and Rosie Kneafsey contributed to study concept and design and acquisition of funding. Shea Palmer contributed to acquisition of the data, analysis of the data, and statistical expertise. All authors (Shea Palmer, Amanda Rodrigues Amorim Adegboye, Gareth Hooper, Aanika Khan, Caroline Leech, Amanda Moore, Bhupinder Pawar, Ala Szczepura, Chris Turner, Rosie Kneafsey) contributed to interpretation of the data, drafting of the manuscript, and critical revision of the manuscript for important intellectual content.

## FUNDING INFORMATION

This research was supported by a grant from Health Education England.

## CONFLICT OF INTEREST STATEMENT

AK was employed by Health Education England during the research and was part of the team that awarded the research grant. The other authors declare no conflicts of interest.

## ETHICS STATEMENT

Ethical approval was received from Coventry University Ethics Service (Reference P124919).

## Supporting information


**Data S1.** Copy of the online survey.

**Data S2.** Additional survey participant demographics.

**Data S3.** List of leadership courses identified by ‘Other training’ survey responders (*n* = 90).

**Data S4.** Summary of responses specific to EMLeaders Training. *Responders could select more than one option for these questions, so the total number of responses has been reported [but please note that the % figures reported are as a proportion of the *n* = 177 responders who undertook EMLeaders Training].

## References

[1] Larsen T , Beier‐Holgersen R , Meelby J , Dieckmann P , Østergaard D . A search for training of practising leadership in emergency medicine: a systematic review. Heliyon. 2018;4(11):e00968. 30761367 10.1016/j.heliyon.2018.e00968 PMC6286301

[2] Bhanja A , Hayirli T , Stark N , Hardy J , Peabody CR , Kerrissey M . Team and leadership factors and their relationship to burnout in emergency medicine during COVID‐19: a 3‐wave cross‐sectional study. J Am Coll Emerg Physicians Open. 2022;3(4):e12761. 35782348 10.1002/emp2.12761 PMC9245504

[3] Lateef F . Grace under pressure: leadership in emergency medicine. J Emerg Trauma Shock. 2018;11(2):73‐79. 29937634 10.4103/JETS.JETS_18_18 PMC5994854

[4] Lyons O , George R , Galante JR , et al. Evidence‐based medical leadership development: a systematic review. BMJ Leader. 2021;5:206‐213. 10.1136/leader-2020-000360 37850339

[5] Hansen M , Harrod T , Bahr N , et al. The effects of leadership curricula with and without implicit bias training on graduate medical education: a multicenter randomized trial. Acad Med. 2022;97(5):696‐703. 34966032 10.1097/ACM.0000000000004573 PMC13183455

[6] General Medical Council . National Training Survey 2023 Results. General Medical Council; 2023.

[7] Lin M , Battaglioli N , Melamed M , Mott SE , Chung AS , Robinson DW . (2019) high prevalence of burnout among US emergency medicine residents: results from the 2017 national emergency medicine wellness survey. Ann Emerg Med. 2019;74(5):682‐690. 30879701 10.1016/j.annemergmed.2019.01.037

[8] Darbyshire D , Brewster L , Isba R , Body R , Basit U , Goodwin D . Retention of doctors in emergency medicine: a scoping review of the academic literature. Emerg Med J. 2021;38(9):663‐672. 34083428 10.1136/emermed-2020-210450 PMC8380914

[9] Daniels J , Robinson E , Jenkinson E , Carlton E . Perceived barriers and opportunities to improve working conditions and staff retention in emergency departments: a qualitative study. Emerg Med J. 2024;41(4):257‐265. 38195524 10.1136/emermed-2023-213189 PMC10982618

[10] EMLeaders National Faculty . EMLEADERS FRAMEWORK. Syllabus for Emergency Medicine Leadership: Personal, Professional and Team Leadership to Improve Patient Care. 2021 Accessed July 17 2024. https://rcem.ac.uk/wp‐content/uploads/2021/10/EMLeaders_Framework_vs.3_100521.pdf

[11] Kneafsey R , Moore A , Palmer S , et al. Tailored leadership training in emergency medicine: qualitative exploration of the impact of the EMLeaders programme on consultants and trainees in England. Emerg Med J. 2024;41:543‐550. doi:10.1136/emermed-2023-213868 39009425 PMC11347192

[12] Sharma A , Minh Duc NT , Luu Lam Thang T , et al. A consensus‐based checklist for reporting of survey studies (CROSS). J Gen Intern Med. 2021;36(10):3179‐3187. 33886027 10.1007/s11606-021-06737-1 PMC8481359

[13] Chałupnik M , Atkins S . “Everyone happy with what their role is?”: a pragmalinguistic evaluation of leadership practices in emergency medicine training. J Pragmat. 2020;160:80‐96.

[14] Linden JA , Baird J , Madsen TE , et al. Diversity of leadership in academic emergency medicine: are we making progress? Am J Emerg Med. 2022;57:6‐13. 35462120 10.1016/j.ajem.2022.04.009

[15] Rixon A , Elder E , Bull C , et al. Leadership conceptions of nurses and physicians in emergency care: a scoping review. Int Emerg Nurs. 2024;74:101454. 38677058 10.1016/j.ienj.2024.101454

[16] Dyrbye LN , Shanafelt TD , Gill PR , Satele DV , West CP . Effect of a professional coaching intervention on the well‐being and distress of physicians: a pilot randomized clinical trial. JAMA Intern Med. 2019;179(10):1406‐1414. 31380892 10.1001/jamainternmed.2019.2425 PMC6686971

[17] Fainstad T , Mann A , Suresh K , et al. Effect of a novel online group‐coaching program to reduce burnout in female resident physicians: a randomized clinical trial. JAMA Netw Open. 2022;5(5):e2210752. 35522281 10.1001/jamanetworkopen.2022.10752 PMC9077483

[18] Grant AM , Studholme I , Verma R , Kirkwood L , Paton B , O'Connor S . The impact of leadership coaching in an Australian healthcare setting. J Health Organ Manag. 2017;31(2):237‐252. 28482767 10.1108/JHOM-09-2016-0187

[19] Mcgonagle AK , Schwab L , Yahanda N , et al. Coaching for primary care physician well‐being: a randomized trial and follow‐up analysis. J Occup Health Psychol Assoc. 2020;25(5):297‐314. 10.1037/ocp0000180 32297776

[20] Roberts T , Daniels J , Hulme W , et al. Psychological distress and trauma in doctors providing frontline care during the COVID‐19 pandemic in the United Kingdom and Ireland: a prospective longitudinal survey cohort study. BMJ Open. 2021;11:e049680. 10.1136/bmjopen-2021-049680 PMC8275363 34244282

[21] Rosenman ED , Branzetti JB , Fernandez R . Assessing team leadership in emergency medicine: the milestones and beyond. J Grad Med Educ. 2016;8(3):332‐340. 27413434 10.4300/JGME-D-15-00400.1 PMC4936849

