# Supplementary material for: Leadership training in emergency medicine: A national survey
Source: AEM Educ Train. 2024 Nov 21;8(6):e11047. doi: 10.1002/aet2.11047 (PMC11582086; doi:10.1002/aet2.11047)
Supplement: Supplementary file 1 — Data S1. Copy of the online survey. [file AET2-8-e11047-s004.docx]

**Supplementary File 1. Copy of the online survey.**

**Emergency Medicine Leadership Survey**

**Introduction & Consent**

**EVALUATION OF EMERGENCY MEDICINE LEADERSHIP**

The EMLeaders programme was developed in partnership between The Royal College of Emergency Medicine (RCEM), Health Education England (HEE) and NHS Improvement/England (NHSI/E). The purpose of the programme is to improve the quality of leadership skills being deployed in the Emergency Medicine (EM) operational environment and aims to ensure that those working within the Emergency Department (ED) are:

- more knowledgeable about clinical leadership and how to apply it on the shop floor.
- empowered to make decisions in the workplace and manage the challenging environment of the emergency department.
- supported by the School leadership faculty with their learning and are enabled to feed back personal experiences or concerns.

This brief questionnaire has been developed as part of an independent evaluation of the EMLeaders programme, commissioned by HEE. As part of this evaluation, we are also interested in hearing from those who have undertaken other leadership training and those who have not yet had leadership training. The evaluation is being conducted by a team from the Centre for Healthcare Research at Coventry University.

**PARTICIPANT INFORMATION STATEMENT**

The aim of this questionnaire is to evaluate the EMLeaders programme, other leadership training and the experience of those who have not yet undertaken leadership training.

You have been selected to take part in this questionnaire because you were identified by the RCEM as a member and/or as an attendee at EMLeaders events. Your participation in the survey is entirely voluntary, and you can opt out at any stage by closing and exiting the browser. Please note that data entered up to the point of exiting the browser will still be included in analysis. There are no implications if you do not complete the survey.

If you are happy to take part, please answer the following questions relating to your experience of the programme and its impact. The survey should take approximately 10 minutes to complete.

Your answers will be treated confidentially and the information you provide will be kept anonymous in any research outputs/publications. Your data will be held securely by Professor Shea Palmer on a password-protected Jisc Online Survey account and password-protected Coventry University One Drive account. All data will be held for six years and deleted by 31st December 2027. The evaluation project has been reviewed and approved through the formal Research Ethics procedure at Coventry University.

For further information, or if you have any queries, please contact the lead for this part of the evaluation, Professor Shea Palmer, Professor of Allied Health, [ad6948@coventry.ac.uk](mailto:ad6948@coventry.ac.uk). If you have any concerns that cannot be resolved through the lead, please contact Professor Rosie Kneafsey, Director of the Centre for Healthcare Research, [aa9398@coventry.ac.uk](mailto:aa9398@coventry.ac.uk).

Thank you for taking the time to participate in this survey. Your help is very much appreciated.

I consent to the information I provide being used to evaluate the EMLeaders programme. *Required

- Yes

I have read and understood the above information. I understand that, because my answers will be fully anonymised, it will not be possible to withdraw them from the study once I have completed the survey. I agree to take part in this questionnaire survey. I confirm that I am aged 18 or over. *Required

- Yes

**Occupational Details**

Are you currently working in Emergency Medicine (EM)? * Required

- Yes
- No
- If 'Yes' please specify your role __________

Please select which career grade applies to you *Required

- Consultant
- Trainee ST1
- Trainee ST2
- Trainee ST3
- Trainee ST4
- Trainee ST5
- Trainee ST6
- SAS Doctor (Staff Grade, Associate Specialist and Specialty Doctors)
- Physician Associate
- Advanced Care Practitioner
- Locum Consultant
- Other
- If you selected Other, please specify: __________

Have you been involved with supporting participants on EMLeaders training events? *Required

- Yes
- No

**Screening Question 1**

Have you undertaken EMLeaders training events? *Required

- Yes
- No

**Screening Question 2**

Have you undertaken other external leadership training? *Required

- Yes
- No

**EMLeaders Programme Evaluation**

Within which HEE EM School region(s) did you undertake your EMLeaders training events? (select as many as are relevant) *Required

- East Midlands
- East of England
- Thames Valley
- West Midlands
- Northeast
- Northwest & Mersey
- Yorkshire & Humber
- London
- Kent, Surrey & Sussex
- Peninsula
- Severn
- Wessex
- I’m not sure

In which year did you first undertake EMLeaders training? *Required

- 2021
- 2020
- 2019
- I'm not sure

Which of the following aspects of EMLeaders training have you participated in? (select as many as are relevant) *Required

- Faculty development days
- Face to face study days
- E-learning modules
- Communities of practice
- Integrated into local or regional teaching activities

There are currently 9 EMLeaders modules available on the e-Learning for Health (e-LfH) platform. These may have been delivered in alternative formats (e.g. face-to-face study days) in earlier iterations of the programme. Please select which of these modules/study days you believe you have undertaken (select as many as are relevant). *Required

- Leading Self (core)
- Leading Teams (core)
- Leading Systems (core)
- Leading Service
- Leading Culture
- Leading Change
- Leading Quality
- Leading People
- Leading Strategy (previously called ‘Leading Evaluation’)
- None of these

Have you made a decision not to engage in further EMLeaders training? *Required

- Yes
- No
- If ‘Yes’ please specify why __________

Please read each of the following statements and select the strength of your agreement or disagreement with each. *Required

Please don't select more than 1 answer(s) per row.

Please select at least 16 answer(s).

|  | Strongly agree | Moderately agree | Slightly agree | Slightly disagree | Moderately disagree | Strongly disagree |
| --- | --- | --- | --- | --- | --- | --- |
| I am knowledgeable about clinical leadership |  |  |  |  |  |  |
| I know how to apply clinical leadership on the shop floor |  |  |  |  |  |  |
| I am empowered to make decisions in the workplace |  |  |  |  |  |  |
| I can manage the challenging environment of the ED |  |  |  |  |  |  |
| I am supported by the HEE EM School Faculty with my learning and development as a leader |  |  |  |  |  |  |
| I am enabled to feed back personal experiences or concerns |  |  |  |  |  |  |
| I am positive about my ability to influence the EM work environment |  |  |  |  |  |  |
| I am confident in my decision making |  |  |  |  |  |  |
| I am confident in my leadership |  |  |  |  |  |  |
| I am confident in facilitating teams |  |  |  |  |  |  |
| I have positive wellbeing at work |  |  |  |  |  |  |
| I am enthusiastic about pursuing a career in EM |  |  |  |  |  |  |
| I listen effectively to other people within the ED |  |  |  |  |  |  |
| I can recognise the differing demands within the ED |  |  |  |  |  |  |
| I can adapt to the differing demands within the ED |  |  |  |  |  |  |
| I would recommend the EMLeaders training that I undertook to my peers |  |  |  |  |  |  |

Since taking part in the EMLeaders training… How has your knowledge of leadership in EM changed? *Required __________

Since taking part in the EMLeaders training... How has your confidence and/or competence as a leader changed? *Required __________

Regarding the content and delivery of the EMLeaders training… What worked well? *Required __________

Regarding the content and delivery of the EMLeaders training… What would ideal leadership training look like (content and delivery)? *Required __________

We are very interested in speaking to participants in more detail about their experience of the EMLeaders programme, either 1:1 or as part of a focus group (this will be online via an online forum such as MS Teams or Zoom). If you would be happy to discuss the programme with the evaluation team, please add your details below:

I am happy to be contacted by the evaluation team discuss the EMLeaders programme. *Required

- Yes
- No
- Name / email / telephone __________

**Other Leadership Training Evaluation**

Please specify what other external leadership training you have undertaken *Required __________

Within which HEE EM School region(s) do you currently work? (select as many as are relevant) *Required

- East Midlands
- East of England
- Thames Valley
- West Midlands
- Northeast
- Northwest & Mersey
- Yorkshire & Humber
- London
- Kent, Surrey & Sussex
- Peninsula
- Severn
- Wessex
- I’m not sure

Please read each of the following statements and select the strength of your agreement or disagreement with each.

Please don't select more than 1 answer(s) per row.

Please select at least 16 answer(s).

|  | Strongly agree | Moderately agree | Slightly agree | Slightly disagree | Moderately disagree | Strongly disagree |
| --- | --- | --- | --- | --- | --- | --- |
| I am knowledgeable about clinical leadership |  |  |  |  |  |  |
| I know how to apply clinical leadership on the shop floor |  |  |  |  |  |  |
| I am empowered to make decisions in the workplace |  |  |  |  |  |  |
| I can manage the challenging environment of the ED |  |  |  |  |  |  |
| I am supported by my colleagues with my learning and development as a leader |  |  |  |  |  |  |
| I am enabled to feed back personal experiences or concerns |  |  |  |  |  |  |
| I am positive about my ability to influence the EM work environment |  |  |  |  |  |  |
| I am confident in my decision making |  |  |  |  |  |  |
| I am confident in my leadership |  |  |  |  |  |  |
| I am confident in facilitating teams |  |  |  |  |  |  |
| I have positive wellbeing at work |  |  |  |  |  |  |
| I am enthusiastic about pursuing a career in EM |  |  |  |  |  |  |
| I listen effectively to other people within the ED |  |  |  |  |  |  |
| I can recognise the differing demands within the ED |  |  |  |  |  |  |
| I can adapt to the differing demands within the ED |  |  |  |  |  |  |
| I would recommend the external leadership training that I undertook to my peers |  |  |  |  |  |  |

Since taking part in your external leadership training… How has your knowledge of leadership in EM changed? *Required __________

Since taking part in your external leadership training… How has your confidence and/or competence as a leader changed? *Required __________

Regarding the content and delivery of your external leadership training… What worked well? *Required __________

Regarding the content and delivery of your external leadership training… What would ideal leadership training look like (content and delivery)? *Required __________

**Evaluation**

Within which HEE EM School region(s) do you currently work? (select as many as are relevant) *Required

- East Midlands
- East of England
- Thames Valley
- West Midlands
- Northeast
- Northwest & Mersey
- Yorkshire & Humber
- London
- Kent, Surrey & Sussex
- Peninsula
- Severn
- Wessex
- I’m not sure

Please read each of the following statements and select the strength of your agreement or disagreement with each.

Please don't select more than 1 answer(s) per row.

Please select at least 14 answer(s).

|  | Strongly agree | Moderately agree | Slightly agree | Slightly disagree | Moderately disagree | Strongly disagree |
| --- | --- | --- | --- | --- | --- | --- |
| I am knowledgeable about clinical leadership |  |  |  |  |  |  |
| I know how to apply clinical leadership on the shop floor |  |  |  |  |  |  |
| I am empowered to make decisions in the workplace |  |  |  |  |  |  |
| I can manage the challenging environment of the ED |  |  |  |  |  |  |
| I am enabled to feed back personal experiences or concerns |  |  |  |  |  |  |
| I am positive about my ability to influence the EM work environment |  |  |  |  |  |  |
| I am confident in my decision making |  |  |  |  |  |  |
| I am confident in my leadership |  |  |  |  |  |  |
| I am confident in facilitating teams |  |  |  |  |  |  |
| I have positive wellbeing at work |  |  |  |  |  |  |
| I am enthusiastic about pursuing a career in EM |  |  |  |  |  |  |
| I listen effectively to other people within the ED |  |  |  |  |  |  |
| I can recognise the differing demands within the ED |  |  |  |  |  |  |
| I can adapt to the differing demands within the ED |  |  |  |  |  |  |

If you were to undertake leadership training… What would ideal leadership training look like (content and delivery)? *Required __________

**Demographic Characteristics**

What ethnic group do you identify as?

- Asian/Asian British
- Black/African/Caribbean/Black British
- Mixed/Multiple ethnic groups
- Other ethnic group
- Prefer not to say
- White

What ethnicity do you identify as?

- White English / Welsh / Scottish / Northern Irish / British
- White Irish
- White Gypsy or Irish Traveller
- Any other White background
- White and Black Caribbean
- White and Black African
- White and Asian
- Any other Mixed / Multiple ethnic background
- Indian
- Pakistani
- Bangladeshi
- Chinese
- Any other Asian background
- African
- Caribbean
- Any other Black / African / Caribbean background
- Arab
- Any other ethnic group
- Prefer not to say

What is your sex (a question about gender identity will follow)?

- Male
- Female
- Prefer not to say

Is your gender the same as the sex you were assigned to at birth?

- Yes
- No
- Prefer not to say

What is your gender identify? Please specify

- Man
- Woman
- Non-binary
- Gender fluid
- Prefer not to say
- Prefer to self-describe __________

Do you consider yourself to have a seen or unseen disability? We define disability as an ‘impairment that has a substantial, long-term adverse effect on a person’s ability to carry out normal day-to-day activities’

- Yes
- No
- Prefer not to say

If yes, how would you describe your disability or impairment? Tick all that apply

- Developmental
- Learning
- Mental health
- Physical
- Sensory
- Neurodiverse
- Not applicable
- Prefer not to say
- Other
- If you selected Other, please specify: __________

**End**

Thank you very much for completing this survey
