# Supplementary material for: Leadership training in emergency medicine: A national survey
Source: AEM Educ Train. 2024 Nov 21;8(6):e11047. doi: 10.1002/aet2.11047 (PMC11582086; doi:10.1002/aet2.11047)
Supplement: Supplementary file 2 — Data S2. Additional survey participant demographics. [file AET2-8-e11047-s001.docx]

**Supplementary File 2. Additional survey participant demographics**

| **Question/Response** | **RCEM**  **Membership** | **EMLeaders**  **Training**  **n=177** | **Other**  **Training**  **n=92** | **No**  **Training**  **n=148** | **Total**  **n=417** |
| --- | --- | --- | --- | --- | --- |
| ***“What ethnicity do you identify as?”* p=0.025*** | | | | | |
| Indian | N/A | 28 (15.9%) | 12 (13.2%) | 29 (19.7%) | **69 (16.7%)** |
| Pakistani | N/A | 3 (1.7%) | 6 (6.6%) | 10 (6.8%) | **19 (4.6%)** |
| Bangladeshi | N/A | 0 | 0 | 1 (0.7%) | **1 (0.2%)** |
| Chinese | N/A | 2 (1.1%) | 0 | 3 (2%) | **5 (1.2%)** |
| Any other Asian background | N/A | 6 (3.4%) | 4 (4.4%) | 3 (2%) | **13 (3.1%)** |
| African | N/A | 4 (2.3%) | 4 (4.4%) | 1 (0.7%) | **9 (2.2%)** |
| Caribbean | N/A | 0 | 0 | 1 (0.7%) | **1 (0.2%)** |
| Any other Black/ African/ Caribbean background | N/A | 1 (0.6%) | 0 | 1 (0.7%) | **2 (0.5%)** |
| White and Black Caribbean | N/A | 1 (0.6%) | 0 | 0 | **1 (0.2%)** |
| White and Black African | N/A | 0 | 3 (3.3%) | 4 (2.7%) | **7 (1.7%)** |
| White and Asian | N/A | 1 (0.6%) | 0 | 0 | **1 (0.2%)** |
| Any other Mixed/ Multiple ethnic background | N/A | 1 (0.6%) | 3 (3.3%) | 0 | **4 (1%)** |
| Arab | N/A | 7 (4%) | 2 (2.2%) | 11 (7.5%) | **20 (4.8%)** |
| Any other ethnic group | N/A | 1 (0.6%) | 0 | 3 (2%) | **4 (1%)** |
| Prefer not to say | N/A | 5 (2.8%) | 3 (3.3%) | 8 (5.4%) | **16 (3.9%)** |
| White English/ Welsh/ Scottish/ Northern Irish/ British | N/A | 106 (60.2%) | 45 (49.5%) | 61 (41.5%) | **212 (51.2%)** |
| White Irish | N/A | 2 (1.1%) | 5 (5.5%) | 2 (1.4%) | **9 (2.2%)** |
| White Gypsy or Irish Traveller | N/A | 0 | 0 | 1 (0.7%) | **1 (0.2%)** |
| Any other White background | N/A | 8 (4.5%) | 4 (4.4%) | 8 (5.4%) | **20 (4.8%)** |
| ***“Is your gender the same as the sex you were assigned to at birth?”*** p=0.417 | | | | | |
| Yes | N/A | 168 (95.5%) | 89 (96.7%) | 139 (94.6%) | **396 (95.4%)** |
| No | N/A | 0 | 0 | 2 (1.4%) | **2 (0.5%)** |
| Prefer not to say | N/A | 8 (4.5%) | 3 (3.3%) | 6 (4.1%) | **17 (4.1%)** |
| ***“What is your gender identity?”*** p=0.387 | | | | | |
| Man | N/A | 90 (52%) | 58 (63%) | 87 (59.6%) | **235 (57.2%)** |
| Woman | N/A | 75 (43.4%) | 29 (31.5%) | 50 (34.2%) | **154 (37.5%)** |
| Non-binary | N/A | 0 | 0 | 0 | **0** |
| Gender fluid | N/A | 0 | 0 | 1 (0.7%) | **1 (0.2%)** |
| Prefer not to say | N/A | 8 (4.6%) | 5 (5.4%) | 8 (5.5%) | **21 (5.1%)** |
| Prefer to self-describe | N/A | 0 | 0 | 0 | **0** |
| ***“If yes, how would you describe your disability or impairment? Tick all that apply”* p=0.011*** | | | | | |
| Developmental | N/A | 0 | 0 | 0 | **0** |
| Learning | N/A | 5 (4.2%) | 3 (4.8%) | 2 (2.1%) | **10 (3.6%)** |
| Mental health | N/A | 2 (1.7%) | 4 (6.5%) | 1 (1.1%) | **7 (2.5%)** |
| Physical | N/A | 0 | 5 (8.1%) | 3 (3.2%) | **8 (2.9%)** |
| Sensory | N/A | 2 (1.7%) | 0 | 1 (1.1%) | **3 (1.1%)** |
| Neurodiverse | N/A | 6 (5%) | 0 | 2 (2.1%) | **8 (2.9%)** |
| Not applicable | N/A | 99 (82.5%) | 43 (69.4%) | 80 (84.2%) | **222 (80.1%)** |
| Prefer not to say | N/A | 6 (5%) | 5 (8.1%) | 6 (6.3%) | **1 (6.1%)** |
| Other | N/A | 0 | 2 (3.2%) | 0 | **2 (0.7%)** |

**Additional survey participant demographics.** EM = Emergency Medicine; N/A = Not Applicable; RCEM = Royal College of Emergency Medicine. *Statistically significant difference between groups (Pearson Chi-Square, p<0.05).
