# Supplementary material for: Leadership training in emergency medicine: A national survey
Source: AEM Educ Train. 2024 Nov 21;8(6):e11047. doi: 10.1002/aet2.11047 (PMC11582086; doi:10.1002/aet2.11047)
Supplement: Supplementary file 3 — Data S3. List of leadership courses identified by ‘Other training’ survey responders (n = 90). [file AET2-8-e11047-s003.docx]

**Supplementary File 3.** **List of leadership courses identified by ‘Other training’ survey responders (n=90)**

| **Participant Number** | **Type** | **Response (verbatim)** |
| --- | --- | --- |
| 1 | Health | Trust level training at UHBW Bristol |
| 8 | Health | Medical leader in practice  Organised by AQUA |
| 13 | Health | Glass lift leadership training -female Dr's leadership programme I think funded by HEE |
| 14 | Health | Trust course  Flow coaching academy |
| 16 | Health | I had training with world academy of medical leadership UK |
| 18 | Health | PGC in Clinical Leadership  Academic wales Leadership Programme |
| 20 | Health | NHS Senior clinical leadership,  eCLIPS |
| 25 | Health | Middle-grade/Tier 4 Development masterclasses organised by my Local ED |
| 29 | Health | Trust local leadership development programme |
| 30 | Health | NHS Leadership programme |
| 31 | Health | Internal leadership course for London North West Hospitals NHS Trust |
| 32 | Health | Management and Leadership training - HEE online modules  EM Leadership modules(RCEM) - HEE online modules |
| 40 | Health | Fit to Lead BAMM  LEAN  fundamentals of leadership  Faculty of medical leadership and management meetings |
| 41 | Health | E learning course on medical leadership. |
| 43 | Health | UHCW trust leadership programme- senior staff |
| 45 | Health | Courses for leadership & management offered to SAS doctors by the trust? |
| 56 | Health | HENW module in clinical leadership in ST6 |
| 57 | Health | BMA leadership event |
| 66 | Health | Lean leadership  Currently taking part.  In trust training |
| 76 | Health | BMA |
| 77 | Health | NHS Leadership Academy award level 5 |
| 78 | Health | Attended regional EM leaders workshops |
| 79 | Health | Leadership training at end of registrar training. Ongoing coaching |
| 80 | Health | Online Medical Leadership and management distance learning course |
| 89 | Health | AQUA |
| 7 | Military | Military |
| 12 | Military | Military - general staff induction course. |
| 17 | Military | Regular Army Commissioning course, Royal Military Academy Sandhurst |
| 33 | Military | Royal Military College Sandhurst |
| 48 | Military | Leadership and Management Programme (NHS Education for Scotland)  Royal Navy: Naval Analysis Course, Junior Officer Leadership course 2 |
| 61 | Military | Regular Army Commissioning Course, Royal Military Academy Sandhurst |
| 87 | Military | I am an officer in the Army reserves which includes 2 weeks at Sandhurst, classed as a leadership academy - obviously with a focus on military leadership. |
| 3 | University | Executive Master's in Medics Leadership, Bayes Business School, City, University of London with additional Level 7 Qualification in leadership & management, Chartered Management Institute. |
| 4 | University | Oxford courses |
| 6 | University | Oxford training course |
| 10 | University | PGCert University of Lancaster  AQUA |
| 11 | University | Leadership and Management course arranged for SAS doctors at my trust  Professional & Generic skills course from Plymouth University |
| 15 | University | Leadership and innovation module provided by the University of the West of England |
| 21 | University | Leadership programme with Imperial business school |
| 22 | University | Masters Educational Leadership |
| 23 | University | MBA module. others I’ve forgotten |
| 24 | University | Keele leadership course, HEA fellowship training |
| 26 | University | MSc leadership module |
| 27 | University | Leadership and mentoring modules within my MA |
| 36 | University | Leadership and Management in Health  University of Washington professional development course |
| 44 | University | Leadership BSc module. Liverpool John Moore's |
| 46 | University | Christ Church University Leadership module at Masters level  East Kent hospitals NHS in-hospital course - Clinical Leadership |
| 49 | University | Keele leadership course |
| 52 | University | Within MSc pathway - Module on Leadership |
| 55 | University | MBA |
| 58 | University | High impact leadership Training at Cambridge university |
| 65 | University | Leadership and Management in Health course at the University of Washington |
| 69 | University | Post grad certificate in health service management |
| 70 | University | Leadership Masterclass at Teesside University |
| 82 | University | Certificate of Leadership and Management in health and social care (University of West England) |
| 86 | University | Keele university |
| 2 | Various | Just completed the leadership plus fellowship |
| 5 | Various | Online training |
| 9 | Various | Kings fund |
| 19 | Various | Online via e-LfH & multiple Webinars & Study days |
| 28 | Various | Leadership course |
| 34 | Various | Multiple leadership roles including appraisal and organ donation leadership roles. |
| 35 | Various | Kings Fund Senior Clinical Leaders Programme. |
| 37 | Various | I don't really remember. Some Oxford course. |
| 38 | Various | With my supervising Consultants. |
| 39 | Various | Leadership workshops and courses. |
| 42 | Various | 1:1 leadership coaching |
| 47 | Various | Course |
| 50 | Various | One to one coaching |
| 51 | Various | Do not remember |
| 53 | Various | Management and leadership course by ISC Medical |
| 54 | Various | Leadership course |
| 59 | Various | Kings fund |
| 60 | Various | Private course |
| 62 | Various | FMLM |
| 63 | Various | Leadership one day workshop in EUSEM  Eclipse leadership program |
| 64 | Various | Leading an Empowered Organisation course |
| 67 | Various | On line modules BMJ Learning |
| 68 | Various | FQIM |
| 71 | Various | ULead |
| 72 | Various | Coursera website |
| 73 | Various | ILM leadership certificate with Cornwall County Council |
| 74 | Various | E-LfH |
| 75 | Various | Em leadership training programme in India |
| 81 | Various | Management course, it covers leadership related matters |
| 83 | Various | Leading an empowered organisation |
| 84 | Various | eCLIPS |
| 85 | Various | Local Leadership course |
| 88 | Various | ETC, December 2021  Nurse leadership course 2015 |
| 90 | Various | local NIMDTA event |

**List of leadership courses identified by survey responders (n=90).** Column 2 (‘Type’) is a categorisation of the type of leadership training, summarised as follows: Health (n=25) = training programmes delivered by NHS, individual Trusts, etc (including AQUA); Military (n=7) = Various military training programmes; University (n=24) = University training programmes; Various (n=34) = Other training programmes. AQUA = Advancing Quality Alliance; BAMM = British Association of Medical Managers; BMA = British Medical Association; BMJ = British Medical Journal; BSc = Bachelor of Science; eCLIPS = embedded Clinical Leadership in Practice Scheme; ED = Emergency Department; EM = Emergency Medicine; E-LfH = E-learning for Health; ETC = European Trauma Course; EUSEM = European Society for Emergency Medicine; FMLM = Faculty of Medical Leadership and Management; FQIM = Fellowship in Quality Improvement; HEA = Higher Education Academy; HEE = Health Education England; HENW = Health Education North West; ILM = Institute of Leadership and Management; ISC Medical = Interview Skills Consulting Ltd.; LEAN = Lean Enterprise Institute; MA = Master of Arts; MBA = Master of Business Administration; MSc = Master of Science; NHS = National Health Service; PGC/PGCert = Postgraduate Certificate; UHBW = University Hospitals Bristol and Weston NHS Foundation Trust; UHCW = University Hospitals Coventry and Warwickshire NHS Trust; NIMDTA = Northern Ireland Medical & Dental Training Agency; RCEM = Royal College of Emergency Medicine; SAS = Specialty and Associate Specialist; ST6 = Specialty Trainee 6.
