# Supplementary material for: Leadership training in emergency medicine: A national survey
Source: AEM Educ Train. 2024 Nov 21;8(6):e11047. doi: 10.1002/aet2.11047 (PMC11582086; doi:10.1002/aet2.11047)
Supplement: Supplementary file 4 — Data S4. Summary of responses specific to EMLeaders Training. *Responders could select more than one option for these questions, so the total number of responses has been reported [but please note that the % figures reported are as a proportion of the n = 177 responders who undertook EMLeaders Training]. [file AET2-8-e11047-s002.docx]

**Supplementary File 4. Summary of responses specific to EMLeaders Training. ***Responders could select more than one option for these questions, so the total number of responses has been reported [but please note that the % figures reported are as a proportion of the n=177 responders who undertook EMLeaders Training].

| ***“Within which HEE EM School region(s) did you undertake your EMLeaders training events?” (n=184)**** | |
| --- | --- |
| East Midlands | 11 (6.2%) |
| East of England | 10 (5.6%) |
| Thames Valley | 10 (5.6%) |
| West Midlands | 20 (11.3%) |
| Northeast | 4 (2.3%) |
| Northwest & Mersey | 33 (18.6%) |
| Yorkshire & Humber | 20 (11.3%) |
| London | 33 (18.6%) |
| Kent, Surrey & Sussex | 10 (5.6%) |
| Peninsula | 8 (4.5%) |
| Severn | 8 (4.5%) |
| Wessex | 13 (7.3%) |
| I’m not sure | 4 (2.3%) |
| ***“In which year did you first undertake EMLeaders training?”*** | |
| 2021 | 66 (37.3%) |
| 2020 | 25 (14.1%) |
| 2019 | 67 (37.9%) |
| I’m not sure | 19 (10.7%) |
| ***“Which of the following aspects of EMLeaders training have you participated in? (n=403)**** | |
| Faculty development days | 51 (28.8%) |
| Face-to-face study days | 112 (63.3%) |
| E-learning modules | 129 (72.9%) |
| Communities of practice | 23 (13%) |
| Integrated into local or regional teaching activities | 88 (49.7%) |
| ***“There are currently 9 EMLeaders modules available on the e-Learning for Health (e-LfH) platform. These may have been delivered in alternative formats (e.g., face-to-face study days) in earlier iterations of the programme. Please select which of these modules/study days you believe you have undertaken” (n=696)**** | |
| Leading Self (core) | 135 (76.3%) |
| Leading Teams (core) | 135 (76.3%) |
| Leading Systems (core) | 104 (58.8%) |
| Leading Service | 53 (29.9%) |
| Leading Culture | 63 (35.6%) |
| Leading Change | 49 (27.7%) |
| Leading Quality | 48 (27.1%) |
| Leading People | 58 (32.8%) |
| Leading Strategy (previously called ‘Leading Evaluation’) | 29 (16.4%) |
| None of these | 22 (12.4%) |
| **“Have you made a decision not to engage in further EMLeaders training?”** | |
| Yes | 20 (11.3%) |
| No | 157 (88.7%) |
